# Mixed acinar-endocrine carcinoma of the pancreas: a case report and review of the literature

**DOI:** 10.1186/1757-1626-2-6481

**Published:** 2009-04-28

**Authors:** Maria A Kyriazi, Nikolaos Arkadopoulos, Vaia K Stafyla, Anneza I Yiallourou, Nikolaos Dafnios, Theodosios Theodosopoulos, Evi Kairi-Vassilatou, Vassilios Smyrniotis

**Affiliations:** 1 2nd Department of Surgery, Areteion Hospital, University of Athens, Greece; 2 Department of Pathology, Areteion Hospital, University of Athens, Greece

## Abstract

### Introduction

Pancreatic tumors usually display either a ductal, an acinar or an endocrine differentiation. Mixed exocrine and endocrine pancreatic tumors are extremely rare. There have been a few reports of the rare entity of mixed acinar-endocrine carcinoma of the pancreas, where the endocrine cells represent more than 30% of the tumor. We herein describe a case of such a pancreatic tumor in an asymptomatic patient.

### Case presentation

A 74-year-old male patient with no evident clinical symptoms was referred for surgical resection of a large mass located on the pancreatic head, which was confirmed by an abdominal U/S, CT and MRI. FNA of the mass under endoscopic ultrasound guidance showed the cytology specimen to comprise of cells with morphological and immunohistochemical characteristics of endocrine pancreatic neoplasms. The patient underwent a modified Whippleâ€™s procedure and his post-operative course was uneventful. Pathological examination of the tumor revealed a mixed acinar-endocrine carcinoma of the pancreas.

### Conclusion

Mixed tumors of the pancreas are extremely rare and their clinical features and pathogenesis remain unclear. The endocrine component seems to influence their prognosis favorably. Therefore, aggressive surgical therapy remains the only well established line of treatment for these tumors. Further accumulation of clinical cases will help clarify the clinical course and the optimal therapy for these unusual tumors.

## Introduction

Most pancreatic tumors arise as a single cell type, either from the endocrine of exocrine pancreas, and can be categorized into three types: ductal, acinar or endocrine. [1]-[3] Acinar cell carcinoma (ACC) is a rare clinical entity, accounting for only 1% of pancreatic exocrine tumors. [4] Although it is well established that one-third of acinar tumors may express neuroendocrine markers [5], their endocrine component is usually limited to a few scattered cells. When the endocrine cells exceed 25%-30% of the tumor, the lesion is categorized as a mixed acinar-endocrine carcinoma (MAEC). Since 1982, when Ulich et al described this entity for the first time, less than 20 cases have been reported in the literature [6,7]. We report a case of a 74-year-old man who underwent a Whippleâ€™s procedure for a large tumor of the pancreatic head that proved to be a mixed acinar-endocrine carcinoma.

## Case presentation

**Figure 1:**
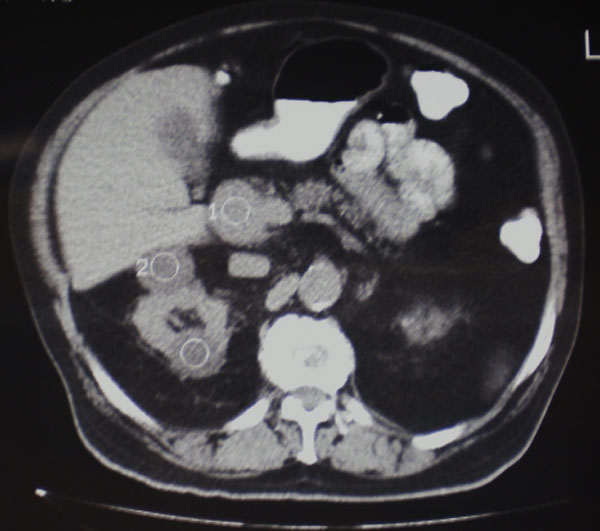
**CT scan image showing a large mass of 6**.8 cm in diameter in direct contact with the head of the pancreas and the inferior vena cava (marked as 1 on the figure - marked as 2 & 3 coexisting simple kidney cysts).

A 74-year-old, white male of Greek origin, was admitted in our hospital in December 2008 in order to be treated for a large mass located in the head of the pancreas. The patient had been originally subjected to a routine blood check up, which revealed mildly elevated transaminase levels. He was then referred for an abdominal U/S and eventually for a CT scan on an outpatient basis. Both revealed a large, well circumscribed mass of 6.8 cm in diameter, which seemed to be in direct contact with the head of the pancreas and the inferior vena cava (Figure 1). A subsequent MRI of the abdomen showed the mass to measure about 7.3 cm, to have irregular borders and to be located posterolaterally to the pancreatic head. The inferior vena cava was located directly behind the mass, and seemed to be slightly displaced by the tumor. The portal vein was located directly above the mass. Both these vessels did not appear to be directly invaded by the mass.

Endoscopic ultrasound scan was performed which allowed FNA of the mass. The cytology revealed cells morphologically and immunogistologically indicative of a neuroendocrine pancreatic neoplasm. Subsequently, the patient was referred to our hospital for further surgical and possible antiproliferative therapy.

On physical examination the patient was found to manifest periodic choreo - athetosic movements that had appeared in the 3 months that preceded the diagnosis of his pancreatic tumor, to together with polyarthralgia of his limbsâ€™ large joints. The rest of his neurological clinical examination as well as an MRI of the brain were normal. Serum amylase, bilirubin and transaminase levels and all serum tumor markers were within normal range.

**Figure 2:**
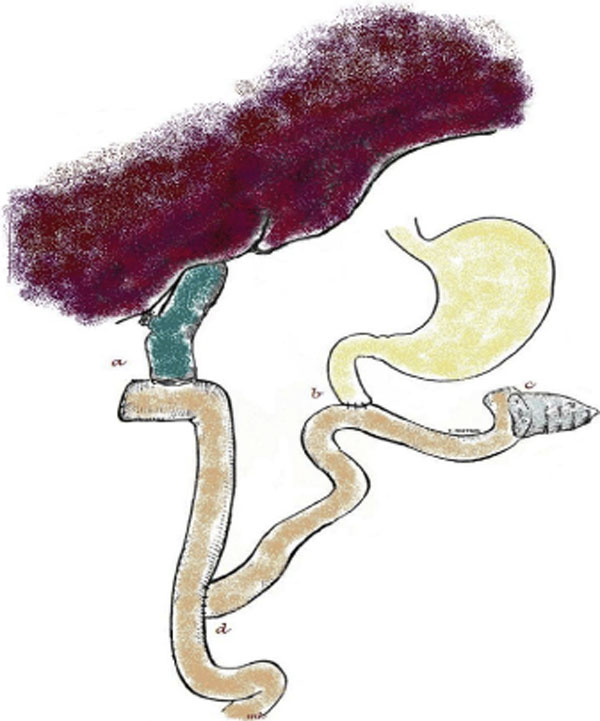
**Diagrammatic depiction of the final Roux-en-Y reconstruction (following a modified Whippleâ€™s procedure). **a. Hepaticojejunostomy, **b.** Duodenojejunostomy, **c.** end-to-side duct to mucosa pancreatojejunostomy, **d.** end-to-side jejunojejunostomy.

The patient underwent surgical resection of the tumor by means of a modified Whippleâ€™s procedure and Roux-n-Y reconstruction of the gastrointestinal tract (Figure 2). His recovery was uneventful and he was discharged from the hospital 10 days after the operation.

**Figure 3:**
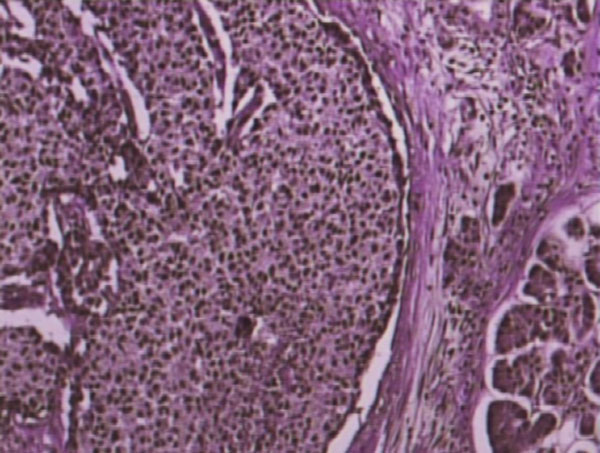
**Histological section showing both the acinar and neuroendocrine features of the pancreatic tumor (Hematoxylin - eosin, Ã— 120)**.

**Figure 4:**
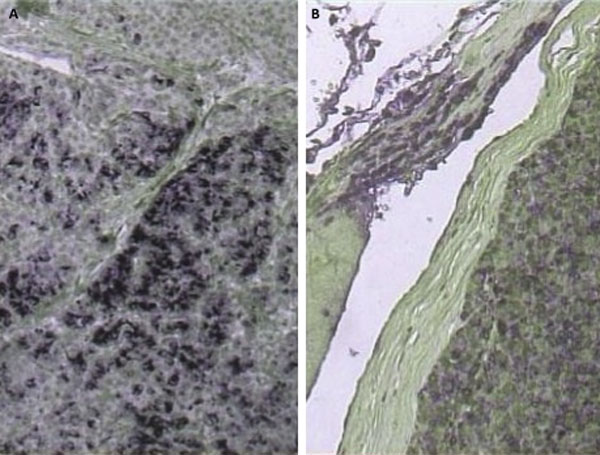
****A**.** Histological section of the pancreatic tumor, showing intense focal positive reaction to synaptophysin (immunostain, Ã— 120), **B.** Histological section of the pancreatic tumor showing diffuse positive immunoreaction to Î±1-antitrypsin (immunostain, Ã— 120).

Pathological examination of the surgical specimen revealed a large pancreatic tumor, located in the head of the pancreas, measuring 12 Ã— 9 Ã— 6 cm. Microscopical examination showed a malignant neoplasm with morphological features of a mixed acinar-endocrine pancreatic tumor. The tumor was multilobular, well circumscribed and fully encapsulated. Numerous neoplastic emboli were present in the vascular and lymphatic channels of the adjacent pancreatic as well as peripancreatic fatty tissue and in the vessels of the outer muscular layer of the duodenum, which was resected en bloc with the tumor. Moreover, 9 out of 10 resected lymph nodes harboured metastases of the neoplasm. Immumohistochemistry revealed (Figures 3,4) intense positivity for chromogranin (25%), mild to intense positivity for synaptophysin (20%), negativity for NSE, C56, CK7, positivity for Î±1-antitrypsin (80%), expression of Ki-67 in 80% and no expression of p53.

The patient remains well 3 months after his operation, with no evidence of recurrence. He also has a complete remission of his neurological symptoms.

## Discussion

The pancreas is well known to be composed from two entirely different components, that is the exocrine and the endocrine pancreas. The exocrine pancreas comprises mainly from duct cells and secondly from acinar cells, while the endocrine pancreas consists of islet cells. Pancreatic tumors usually arise from one of these cell types, resulting in either ductal adenocarcinomas (>75% of pancreatic tumors), neuroendocrine carcinomas (about 7% of pancreatic tumors) and acinar cell carcinomas (about 1%)[8].

Acinar cell tumors show a male predilection, they tend to be larger than ductal adenocarcinomas and they are usually diagnosed between the fifth and the seventh decade of life [4,9]. Their most common location is the head of the pancreas (56%), followed by the tail (36%) and the body (8%). They usually present with non-specific symptoms, such as anorexia, abdominal pain, nausea and vomiting and weight loss. Jaundice is uncommon in ACC, even when they are located in the head of the pancreas. [4,9] Subcutaneous fat necrosis, paniculitis, polyarthralgia and blood eosinophilia may also be noted, when the tumor secretes lipase. [11,12]

Surgical resection is the treatment of choice in these tumors, which are found to demonstrate a higher resectability rate of 64%, compared to that of ductal adenocarcinoma (10-20%) [4]. Although data are limited due to the rarity of these tumors, it appears that patients with acinar cell carcinoma have a slightly better prognosis than those suffering from ductal adenocarcinomas. However, although 1-year survival is just over 50%, ACC remains a very aggressive tumor with an overall 5-year survival of 5.9% [4,9]. Moreover, it has been suggested that ACC is more aggressive than endocrine neoplasms. The recurrence rate even after complete surgical resection is high, suggesting that there are micrometastases present even when the tumor appears to be well localized. However, the results of adjuvant chemotherapy and radiotherapy have been rather disappointing [7].

It is well known that up to one-third of acinar cell carcinomas may express neuroendocrine markers [5,9], which are usually limited to a few scattered cells. Occasionaly, the endocrine cells may add up to more than 30% of the tumor mass, in which case the neoplasm is called a mixed acinar-endocrine carcinoma (MAEC). Nowadays, many scientists believe that MAEC is a special type of ACC, and its pathogenesis can be explained embryologically. Specifically, the embryonic pancreas is known to develop from the foregut and thereafter forms ducts, acini and islets. Both exocrine and endocrine pancreatic cells are thought to originate from multipotential epithelial cells [1,3].

Mixed acinar-endocrine carcinomas are usually large tumors, located in the head of the pancreas (60% of the cases). They usually become clinically evident in middle-aged patients (mean age 58 years). Like ACC, MAEC has no specific clinical symptoms. Patients complain of jaundice, weight loss, vague abdominal pain and only rarely do they present with an endocrine syndrome [13,14]. Specifically in our case the patient complained of choreo-athetosic movements of recent onset and polyarthralgia that completely regressed after surgery, leading us to speculate about the existence of a possible causal relationship between his neurologic symptoms and the pancreatic tumor. This, however, remains to be proven. The only constant difference that is noted between ACC and MAEC is the number of endocrine cells that they contain. Most of the features, such us histological differentiation, tumor size and location and nuclear p53 expression are comparable [5]. The only difference reported in some series is the gender predominance, which is mainly male for ACC and female in women. However, considering the small number of MAECâ€™s reported in the literature so far (less than 20) this difference may well be incidental [3,5].

It must be noted that most of the cases reported so far refer to tumors that were resected completely, thus allowing an accurate diagnosis. It is impossible to define whether more than 1/3 of a pancreatic tumor cells are neuroendocrine if it can not be resected completely. Therefore, it may well be that the true incidence of these tumors is underestimated.

The differentiation of ACC and MAEC from endocrine neoplasms by means of standard histological techniques can prove rather challenging. The presence of cells containing periodic acid-Schiff positive granules which are immunohistochemically positive for pancreatic enzymes (such as trypsin, chymotrypsin and lipase) as well as for endocrine markers (chromogranin and synaptophysin), together with evidence of endocrine hormones are indicative of tumor differentiation toward both acinar and endocrine cell carcinoma [7]. Furthermore, it is easily understood that FNA cytology of these tumors may be misleading as to their true histologic nature, as was the case with our patient, as well. The malignancy potential can be demonstrated by tumor necrosis, numerous mitotic figures and the presence of nuclear atypia with prominent nucleoli.

Regarding the prognosis of patients operated on for MAEC, one report confirmed that out of the first 11 cases ever reported 4 patients died after 5 to 24 months of diagnosis, while 6 were reported to be disease free 4 to 72 months following the primary diagnosis [3]. There has also been a report of a patient with a MAEC that become evident with symptoms of Zollinger-Ellison syndrome, who died from disseminated carcinomatosis 24 years after being operated on for the primary tumor [13]. Mean survival after surgical resection of the primary tumor has been calculated at 10.5 months [7].

Due to the small number of cases reported to date and the relative lack of large scale follow-up data after operation, there are still many controversies on the matter of the optimal course of treatment. Surgery is the first line of treatment in all the cases that the tumor proves to be resectable, however there have been reports of patients benefiting from surgical tumor debulking and local and systemic antiproliferative therapy. It has been stated that the presence of a neuroendocrine component may be related to a more favorable outcome [15]. The rarity of this pancreatic tumor makes it difficult to establish such a relationship without a large multicentric study consisting of a large number of patients. Therefore, aggressive surgery should continue to be the gold standard of their treatment, since it has been the one to demonstrate satisfactory long-term survival results.

## Consent

Written informed consent was obtained from the patient for publication of this case report and accompanying images. A copy of the written consent is available for review by the journalâ€™s Editor-in-Chief.

## Competing interests

The authors declare that there are no conflicts of interests.

## Authorsâ€™ contributions

**MK:** designed and drafted the manuscript.

**NA:** responsible for critical revision of scientific content.

**VS:** assisted in drafting the manuscript.

**AY:** assisted in drafting the manuscript and acquisition of data.

**ND:** made substantial contributions to manuscript conception and acquisition of data.

**TT:** made substantial contributions to manuscript conception and acquisition of data.

**EKV:** performed histopathological and immunohistochemical analyses and contributed to the pathology report content.

**VS:** the surgeon, approved the final version of the manuscript for publication.

All authors read and approved the final version of the manuscript.
